# Discovery and Development of Anti-HBV Agents and Their Resistance

**DOI:** 10.3390/molecules15095878

**Published:** 2010-08-27

**Authors:** Kyun-Hwan Kim, Nam Doo Kim, Baik-Lin Seong

**Affiliations:** 1 Department of Pharmacology, School of Medicine, and Center for Cancer Research and Diagnostic Medicine, IBST, Konkuk University, Seoul 143-701, Korea; 2 Research Institute of Medical Sciences, Konkuk University, Seoul 143-701, Korea; 3 R&D Center, Equispharm Inc., 11F Gyeonggi Bio-Center, 864-1 Iui-Dong, Yeongtong-gu, Suwon-Shi, Gyeonggi-Do 443-766, Korea; 4 Department of Biotechnology, College of Life Science and Biotechnology, Yonsei University, Seoul 120-749, Korea; 5 Translational Research Center for Protein Function Control, Yonsei University, Seoul 120-749, Korea

**Keywords:** hepatitis B virus, antiviral agent, drug development, drug resistance, modeling, rational drug design

## Abstract

Hepatitis B virus (HBV) infection is a prime cause of liver diseases such as hepatitis, cirrhosis and hepatocellular carcinoma. The current drugs clinically available are nucleot(s)ide analogues that inhibit viral reverse transcriptase activity. Most drugs of this class are reported to have viral resistance with breakthrough. Recent advances in methods for *in silico* virtual screening of chemical libraries, together with a better understanding of the resistance mechanisms of existing drugs have expedited the discovery and development of novel anti-viral drugs. This review summarizes the current status of knowledge about and viral resistance of HBV drugs, approaches for the development of novel drugs as well as new viral and host targets for future drugs.

## Abbreviations

cccDNA: covalently closed circular DNA
FDA: U.S. Food and Drug Administration
GASP: Genetic Algorithm Similarity Program
HBsAg: hepatitis B surface antigen
HBeAg: hepatitis B e antigen
HBV: hepatitis B virus
HBx: HBV X protein
HCC: hepatocellular carcinoma
HCV: Hepatitis C virus
HIV: human immunodeficiency virus
HNF: hepatocyte nuclear factor
RT: reverse transcriptase
WHO: World Health Organization
WHV: woodchuck hepatitis virus

## 1. Introduction

Hepatitis B virus (HBV), the prototype of the *Hepadnaviridae* family, is a small virus harboring only four open reading frames. The genome of HBV comprises a partially double-stranded 3.2-kb DNA. Chronic HBV infection is strongly associated with liver diseases like chronic hepatic insufficiency, cirrhosis, and hepatocellular carcinoma (HCC) [1,2,3,4]. The World Health Organization (WHO) estimates that there are currently 400 million individuals worldwide who are chronically infected with HBV, of whom 100 million eventually will die of chronic liver diseases or HCC [5,6]. Although vaccination and drugs against HBV have been introduced successfully, the level of global chronic infection still calls for development of new drugs for the control of HBV infection. 

Infection with HBV in hepatocytes results in the formation of covalently closed circular DNA (cccDNA) in the nucleus, which is the template transcribed to generate four major RNA species. HBV expresses four major viral antigens: precore/core, surface, polymerase and HBV X protein (HBx). Among these HBV-encoded proteins, viral polymerase is the key protein for genome replication and consists of terminal protein (following a spacer), reverse transcriptase (RT), and RNAse H domains. The RT domain is responsible for the minus strand DNA synthesis, the first step in HBV replication, from the pregenomic RNA template [7,8]. Therefore, RT is a fascinating target for anti-HBV drugs. 

All the clinically available HBV drugs are nucleotide or nucleoside analogues that target the activity of viral RT. All drugs approved as anti-HBV agents are reported to have viral resistance due to specific mutations in the RT domain, which encourage the development of novel anti-HBV agents targeting non-polymerase viral or host proteins. In this review we summarize the current status of anti-HBV agents with their viral resistance and the novel small chemical anti-HBV agents targeting non-polymerase proteins.

## 2. Anti-HBV Drugs

The currently available anti-HBV agents are all nucleotide or nucleoside analogues RT inhibitors. These are summarized in Table 1.

### 2.1. Current anti-HBV drugs

#### 2.1.1. Lamivudine

Lamivudine [(-)2′,3′-dideoxy-3′-thiacytidine, commonly called 3TC, Zeffix^®^) was initially developed for the treatment of human immunodeficiency virus (HIV) infection. Lamivudine is a synthetic nucleoside analogue, with activity against HBV and HIV. It is sequentially phosphorylated to lamivudine triphosphate by cellular kinases. After removal of the diphosphate group, lamivudine 5′-monophosphate is incorporated into the growing viral DNA chain at the 3’-end by HBV polymerase. The incorporation of lamivudine into the viral DNA chain induces premature chain termination due to the absence of 3’-OH, which is essential for chain polymerization. 

**Table 1:** Approved anti-HBV drugs.

Molecule	Structure	Brand name	Mechanism	Company	Year of FDA approval
Lamivudine	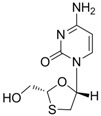	Zeffix, Heptovir, Epivir, and Epivir-HBV	Nucleoside analogue/RT Inhibitor	GlaxoSmithKline	1998 (for adults) and 2000 (for children) in US
Adefovir	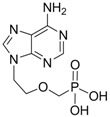	Preveon and Hepsera	Nucleotide analogue/RT Inhibitor	Gilead	2002 (US)
Entecavir	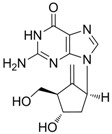	Baraclude	Nucleoside analogue/RT Inhibitor	Bristol Meyers Squibb	2005 (US)
Telbivudine	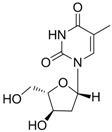	Sebivo (Europe) Tyzeka (US)	Nucleoside analogue/RT Inhibitor	Idenix, Novartis	2006 (US)
Clevudine	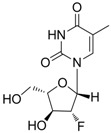	Levovir and Revovir	Nucleoside analogue/RT Inhibitor.	Bukwang Pharm	2006 (KOREA)
Tenofovir	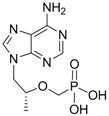	Viread	Nucleotide analogue/RT Inhibitor	Gilead	2008 (US)

*In vitro* antiviral activity of lamivudine against HBV was assessed in HBV DNA-transfected cells and IC_50_ values ranged between 0.01 μM and 3.3 μM, depending on the duration of exposure of cells to lamivudine, the cell model system, and the protocol used [9].

#### 2.1.2. Adefovir Dipivoxil

Adefovir dipivoxil (Hepsera^®, ^9-[2-[[bis[(pivaloyloxy)methoxy]-phosphinyl]-methoxy]ethyl]-adenine) is an acyclic nucleotide analog with activity against HBV. Adefovir was approved by the U.S. Food and Drug Administration (FDA) for use in the treatment of hepatitis B in September 2002 and by the European Union in March 2003. Because adefovir is an analog of adenosine monophosphate, it can be easily phosphorylated to the active metabolite adefovir diphosphate by cellular kinases. Adefovir diphosphate inhibits HBV DNA polymerase by competing with the natural substrate deoxyadenosine triphosphate. The incorporation of adefovir diphosphate into the growing viral DNA causes premature DNA chain termination similar to lamivudine [10]. The inhibition constant (K_i_) for adefovir diphosphate for HBV DNA polymerase is 0.1 μM. *In vitro* antiviral activity of adefovir against HBV was assessed in HBV DNA-transfected human hepatoma cell lines and the IC_50_ values ranged between 0.2 μM and 6.3 μM depending on the assay conditions [9].

#### 2.1.3. Entecavir

Entecavir (Baraclude^®^, 2-amino-1,9-dihydro-9-[(*1S,3R,4S*)-4-hydroxy-3-(hydroxymethyl)-2-methylenecyclopentyl]-6*H*-purin-6-one monohydrate) is a guanosine nucleoside analogue with selective activity against HBV. Entecavir was approved by the U.S. FDA in March 2005 for the treatment of hepatitis B infection. Entecavir is a guanosine nucleoside analogue and is efficiently phosphorylated to the active triphosphate form. By competing with the natural substrate deoxyguanosine triphosphate, entecavir triphosphate functionally inhibits the activities of HBV polymerase. 

Entecavir was more efficacious than other previous agents and had low side effects [11]. Entecavir inhibited HBV DNA synthesis by 50% at a concentration of 3.75 nM in *in vitro* assays [12]. Lamivudine-resistant HBV (rtL180M, rtM204V) was also susceptible to entecavir treatment with high efficacy *in vitro* [13]. Clinical trials revealed that entecavir was superior compared to lamivudine *in vivo* for both hepatitis B e antigen (HBeAg)-positive and HBeAg-negative patients [14,15]. Chronic hepatitis B patients who were refractory to lamivudine treatments showed improved virologic and serology outcomes [16,17].

#### 2.1.4. Telbivudine

Telbivudine [Tyzeka^®^, LdT, 1-(2-deoxy-β-L-ribofuranosyl)-5-methyluracil)] is a synthetic thymidine nucleoside analogue with activity against HBV. Telbivudine is the unmodified L-isomer of the naturally occurring nucleoside, thymidine; therefore, phosphorylation to the active telbivudine triphosphate form by cellular kinases is easily accomplished. The telbivudine 5'-triphosphate eventually inhibits HBV DNA polymerase by competing with the natural substrate, thymidine 5'-triphosphate. Incorporation of telbivudine 5'-triphosphate into replicating HBV DNA causes premature DNA chain termination. *In vitro* antiviral activity of telbivudine against HBV replication was assessed in HBV-stable cell line, hepG2 2.2.15 human hepatoma cells, and the IC_50_ values were around 0.19 μM [18]. Telbivudine was not active against HIV-1 at concentrations of up to 100 μM *in vitro*. Clinical trials have shown that telbivudine is superior to lamivudine or adefovir in patients with chronic hepatitis B [19,20,21].

#### 2.1.5. Clevudine

Clevudine (Levovir^®^, 1-(2-deoxy-2-fluoro-β-arabinofuranosyl)thymine, also known as L-FMAU), is a pyrimidine analog with potent antiviral activity against HBV [22]. Like other nucleoside analogues, clevudine inhibits HBV polymerase by competing with the natural substrate, thymidine. Clevudine has been approved in Korea and the Philippines for the treatment of chronic hepatitis B patients and is now under Phase 3 clinical studies in China. Clevudine inhibits the DNA-dependent DNA activity of HBV polymerase as well as reverse transcription and priming [23,24].

The *in vitro* antiviral activity of clevudine against HBV was assessed in HBV DNA-transfected human hepatoma cells and the IC_50_ value was 0.9 μM [9]. Phase III clinical trial results showed that clevudine therapy for 24 weeks has a potent and sustained antiviral effect in both HBeAg-positive and -negative chronic hepatitis B patients [25,26]. 

#### 2.1.6. Tenofovir

Tenofovir disoproxil fumarate (Viread^®^, 9-[(R)-2-[[bis[[(isopropoxycarbonyl)oxy]methoxy]-phosphinyl]methoxy]propyl] adenine fumarate) is an acyclic nucleotide analog with activity *in vitro* against retroviruses, including HIV-1, HIV-2, and HBV. Tenofovir disoproxil fumarate is an orally bioavailable ester prodrug of tenofovir (also known as PMPA). Tenofovir is a methyl derivative of adefovir and shows a very similar mechanism of action and antiviral resistance pattern to adefovir. Therefore, like adefovir, tenofovir exhibits antiviral activity against lamivudine-resistant HBV *in vivo* and *in vitro* [27,28,29]. 

Tenofovir was approved by the U.S. FDA in October 2001 for the treatment of HIV and in August 2008 for the treatment of chronic hepatitis B. Like adefovir, tenofovir is rapidly metabolized intracellularly into the active metabolite, tenofovir diphosphate, by cellular kinases. Tenofovir diphosphate inhibits HBV DNA polymerase RT by competing with the natural substrate, deoxyadenosine triphosphate, causing the termination of the growing DNA chain since it lacks a 3' hydroxyl group. The Ki for viral DNA polymerase is 0.02 μM, 5-fold lower than the K_i_ of adefovir. 

*In vitro* antiviral activity of tenofovir against HBV replication was assessed using HBV-transfected human hepatoma cell lines and the IC_50_ value was 2.5 μM [9]. Phase III clinical trial results showed that tenofovir therapy for 48 weeks at a daily dose of 300 mg had superior antiviral efficacy with a similar safety profile compared with adefovir dipivoxil at a daily dose of 10 mg [30].

### 2.2. Adverse effects of current HBV drugs

The major adverse effects of long-term administration of nucleotide or nucleoside RT inhibitors are nephrotoxicity and myopathy [31,32]. Nephrotoxicity is characterized by gradual increases in serum creatinine and decreases in serum phosphorus due to the inhibition (or toxicity) of kidney function. Adefovir and tenofovir (structural analogue of adefovir) are reported to be associated with nephrotoxicity at higher doses in HIV-infected patients and in chronic hepatitis B patients [31,33,34]. In the case of adefovir, the FDA did not approve the drug due to concerns about the severity and frequency of kidney toxicity when dosed at 60 or 120 mg for the treatment of HIV infection [35]. However, Gilead Sciences continued its development for HBV treatment, for which it is effective at a much lower dose of 10 mg. Because the long-term administration of adefovir may increase the overall risk of nephrotoxicity, it is important to monitor renal function for all patients during treatment with adefovir and tenofovir.

The major human DNA polymerases include DNA polymerases alpha, beta, gamma, delta and epsilon. Most of the nucleotide or nucleoside analogues are weak inhibitors of mammalian alpha-, beta-, and gamma-DNA polymerases [36]. This inhibition is likely related to drug-induced adverse effects. Among these, DNA polymerase gamma is located in mitochondria whereas other polymerases are found in the nucleus. The mitochondrial DNA polymerase gamma is reported to have both DNA- and RNA-dependent DNA polymerase activity similar to those of viruses such as HBV and HIV. The functional similarity between viral polymerase and mitochondrial DNA polymerase gamma is likely responsible for the nucleoside drug-induced toxicity. DNA polymerase gamma is reported to have higher affinity for nucleotide or nucleoside RT inhibitors. The incorporation of nucleoside inhibitors into the mitochondrial DNA induces premature genome termination, resulting in mitochondrial DNA deletions (see review by references [37,38]).

Myopathy characterized by muscle pain, muscle weakness or muscle tenderness is observed in patients who have received long-term treatment of clevudine and telbivudine [38,39]. Seok *et al*. [39] reported that seven patients who received 8-13 months of clevudine therapy showed severe evidence of myopathy with elevated creatine kinase levels. Muscle biopsies and molecular studies showed that the myopathy is associated with the depletion of mitochondria DNA and myonecrosis, possibly due to the inhibition of polymerase gamma by clevudine. These findings indicate that long-term treatment with structurally similar telbivudine and clevudine (fluoro-telbivudine) may lead to mitochondrial toxicity-mediated myopathy [32].

## 3. Viral Resistance to HBV Drugs

### 3.1. Molecular mechanism of resistance

Like other viral polymerases, the reverse transcriptase of HBV polymerase lacks proofreading activity on the newly synthesized viral genome, resulting in the introduction of random mutations into the progeny HBV DNA. The error rate of HBV polymerase is approximately 1 per 10^5^ to 10^7^ base synthesis due to the error-prone nature of HBV RT [40,41]. The HBV quasispecies produced by the infidelity of HBV RT could account for the emergence of the many natural mutants with point substitutions [42]. Under selection pressure from the host immune system or administration of antiviral agents, these quasispecies can converge into a dominant HBV mutant that can escape the selection pressure. The molecular mechanisms of resistance to nucleos(t)ide analogue drugs and the clinical importance of resistance for chronic hepatitis B have been comprehensively reviewed recently [43,44]. The well established and documented escape mutants against long-term antiviral treatment are as follows. 

#### 3.1.1. Lamivudine resistance

The incidence of resistance during lamivudine treatment increases progressively at rates of 14 to 32% annually, approaching 80% after 48 months of treatment [45]. Genotypic analysis of viral isolates obtained from patients who showed breakthrough of HBV replication while receiving lamivudine enabled the identification of the primary resistance mutations to lamivudine. The element for primary resistance to lamivudine was mapped to mutations in the YMDD motif, a common active site in reverse transcriptase, in the C domain of HBV Polymerase. The rtM204I and rtM204V mutations, resulting in YIDD and YVDD, respectively, have been identified as primary resistance elements to lamivudine treatment [46]. The YMDD mutation is usually accompanied by a compensatory mutation, rtL180M, which enhances the viral replication of replication-defective rtM204I/V mutants [47]. Other compensatory mutations, L80I/V and V173L, in combination with rtM204I/V with or without rtL180M have also been identified [48,49]. *In vitro* drug susceptibility assays showed that mutations that confer resistance to lamivudine decrease the drug sensitivity by 100- to 1000-fold. Lamivudine resistance does not confer cross-resistance to adefovir dipivoxil or tenofovir. The molecular mechanisms of drug resistance have been predicted based on the crystal structure and molecular modeling [50,51].

#### 3.1.2. Adefovir Dipivoxil resistance

The rate of HBV resistance to adefovir is less frequent compared to the resistance to lamivudine. After two years of adefovir monotherapy in patients with HBeAg-negative chronic hepatitis, approximately 2% of patients were reported to have drug resistance. However, the rate of resistance increased considerably to 30% after five years of adefovir monotherapy [52,53]. 

Genotypic analysis has revealed that adefovir resistance is conferred by the substitution of threonine for asparagine at codon 236 (rtN236T) in the D domain, and alanine for threonone or valine at codon 181 (rtA181T/V) in the B domain of HBV polymerase RT [54]. *In vitro* drug susceptibility assays showed that the rtN236T mutation does not affect sensitivity to lamivudine, telbivudine and entecavir; however, rtA181T mutation decreased susceptibility to lamivudine (<10-fold), adefovir (2- to 8-fold) and tenofovir (2- to 3-fold) [55,56].

#### 3.1.3. Entecavir resistance

Entecavir is the most potent of the currently available anti-HBV agents. Resistance to entecavir was not reported in early clinical trials because the incidence of resistance in treatment-naïve patients was very low, less than 1% at 2 years [57]. Long-term monitoring of entecavir resistance has revealed that the resistance rate was only 1.2% even after five years of therapy in treatment-naïve patients [58]. However, when lamivudine-failure patients, who had lamivudine resistance, were treated with entecavir, the incidence of resistance to entecavir dramatically increased to 14% at two years and 51% at five years [58].

The mutations associated with the primary resistance to entecavir are the most complex and have not been fully established in patients. Mutations in the viral polymerase associated with the emergence of entecavir resistance have been mapped to the B domain (rtI169T, rtL180M, rtS184G), C domain (rtM204I/V, rtS202I), and E domain (rtM250V). The lamivudine mutations, rtM204I/V with or without rtL180M, along with other mutations are frequently detected in patients who exhibit entecavir resistance [59]. *In vitro* cell-based drug susceptibility assays also showed that the presence of lamivudine-resistant mutations leads to several hundredfold increases in entecavir resistance. Entecavir resistance does not confer cross-resistance to adefovir dipivoxil and tenofovir [44].

#### 3.1.4. Telbivudine resistance

The cumulative frequency of genotypic resistance to telbivudine in HBeAg-positive patients at 52 and 104 weeks was 5% and 25.1%, respectively, whereas, in HBeAg-negative patients, the resistance incidence was 2.2% and 10.8%, respectively [19,20]. The resistance rate at two years was lower than that of lamivudine (42%) and higher than those of adefovir (3%) and entecavir (<1%) [60]. 

Genotypic analysis from telbivudine-treatment failure isolates showed that the rtM204I/V substitution was associated with virologic failure and rebound. The rtM204I substitution confers the primary resistance to telbivudine treatment and was frequently found with substitutions rtL80I/V and rtL180M. *In vitro* drug susceptibility assays showed that rtM204I, rtL180M + rtM204V, rtI169T/rtM250V and rtT184G/rtS202I mutations exerted strong resistance to telbivudine treatment [61]. Because telbivudine is an L-nucleoside analogue, telbivudine resistance does not confer cross-resistance to adefovir, tenofovir, and entecavir [62].

#### 3.1.5. Clevudine resistance

As an L-nucleoside analogue, clevudine (a fluorinated telbivudine) has a similar resistance profile to lamivudine and telbivudine. Because clevudine was approved very recently, long-term cumulative data on the incidence and genotypic mutations that cause clevudine resistance in chronic hepatitis B patients is not available. 

The rtM204I mutation was identified during week 32 of clevudine therapy in woodchuck HBV [63]. *In vitro* studies using site-directed mutagenesis have also demonstrated that clevudine is not active against HBV harboring a single rtM204I mutant [64,65]. Recently, Kwon *et al.* [9] identified several conserved mutations in RT domain during viral BT from four clevudine-failure patients, with rtM204I being the most common. *In vitro* phenotypic analysis showed that mutation rtM204I was predominantly associated with clevudine resistance, whereas rtL229V was a compensatory mutation for the impaired replication of the rtM204I mutant. They found that a quadruple mutant (rtL129M + rtV173L + rtM204I + rtH337N) conferred greater replicative ability and strong resistance to both clevudine and lamivudine. All of the clevudine-resistant clones were also lamivudine-resistant, but were susceptible to adefovir, entecavir, and tenofovir except for one mutant clone [9].

#### 3.1.6. Tenofovir resistance

Tenofovir is an adefovir analogue, methyl adefovir, presenting a similar mechanism of action and characteristics to adefovir. Tenofovir was approved for chronic hepatitis B treatment very recently; therefore the resistance mutation and resistance rate of tenofovir has not been well studied yet. 

The substitution rtA194T, along with rtL180M + rtM204V, has been found in patients with HIV-HBV co-infection who showed resistance to tenofovir treatment [66]. A recent *in vitro* drug susceptibility assay demonstrated that the rtA194T mutation is associated with partial tenofovir resistance (5- to7-fold increase), regardless of rtL180M + rtM204V mutation, and negatively impacts the replication competence of HBV constructs. Viral replication of rtA194T mutant can be restored to WT levels, if this mutation occurs together with precore or basic core promoter substitutions found in HBeAg-negative hepatitis B [67]. No genotypic substitutions in RT associated with phenotypic resistance to tenofovir were detected during 48-week treatment [30]. Limited *in vivo* and *in vitro* studies on the antiviral activity of tenofovir showed that lamivudine- and adefovir-resistant mutants are susceptible to tenofovir [9,28,29,62].

### 3.2. Molecular modeling study of drug-resistant HBV

The currently available anti-HBV drugs show potent antiviral activity in patients with chronic hepatitis B; however, the resistance and cross-resistance to the drugs is a major obstacle in long-term treatment. Many studies have been conducted to understand the molecular basis of drug resistance, and the mechanistic characterization and molecular modeling of anti-HBV drugs complexed with HBV RT have been reported. Although the three-dimensional X-ray structure of HBV polymerase is not available, its homology model has been reported using the X-ray structure of HIV RT as a template [48,51,68,69,70]. Even though the homology models may not be accurate due to the low sequence homology between the overall HIV and HBV polymerase, the sequence conservation between the RT domains of HIV and HBV polymerase enables molecular modeling of HBV RT [71]. In particular, the residues around the active site that are responsible for recognizing the template-primer or an incoming nucleoside triphosphate are highly conserved. Nucleoside analogue HBV polymerase inhibitors cause chain termination after incorporation into the growing chain in the active site of HBV polymerase and consequently inhibit viral reverse transcriptase. Thus, the HBV homology model structure based on the crystal structure of HIV polymerase serves as a useful guide for understanding the molecular basis of HBV resistance to drugs. 

Molecular dynamics studies on the homology model structure of HBV can provide useful information regarding mutations associated with resistance to inhibitors of HBV polymerase. Daga *et al*. [72] built a stereochemically significant homology model of HBV polymerase and suggested a significant role for conserved Lys 32 residue in HBV RT, which corresponds to Lys 65 in HIV RT, in binding of nucleotides and known HBV RT inhibitors. Their homology model of HBV polymerase had two main differences from previous reports: They aligned the sequence by using the proper match of a conserved Lys residue in HIV-1 RT, which has important salt bridge interactions with the γ-phosphate of the incoming nucleotide. Secondly, they used a different template structure of HIV-1 RT (PDB code: 1T05) that was a higher resolution crystal structure compared to the previously used template (PDB code: 1RTD). Based on this modeling result, they provided an explanation for the various resistant mutants of HBV polymerase and successfully predicted binding conformations of known HBV inhibitors [72].

Das K *et al.* [51] constructed a three-dimensional homology model of the catalytic core of HBV polymerase based on the crystal structure of HIV-1 RT. Molecular modeling studies using the HBV polymerase homology model suggest that steric hindrance between the mutant amino acid side chain and lamivudine or emtricitabine, anti-HBV-drug, could account for the resistance phenotype. Specifically, steric conflict between the Ile or Val at position rt204 in HBV polymerase and the sulfur atom in the oxathiolane ring of lamivudine and emtricitabine is proposed to account for the resistance observed with rtM204I or rtM204V mutation. The effects of the rtL180M mutation, which also occurs near the HBV polymerase active site, appeared to be less direct, potentially involving rearrangement of the deoxynucleoside triphosphate-binding pocket residues.

Sharon *et al*. [70] constructed a homology model structure of HBV polymerase, which is used for minimization, conformational search and induced fit docking followed by binding energy calculation for wild-type and mutant HBV polymerases (rtL180M, rtM204V, rtM204I, rtL180M + rtM204V, rtL180M + rtM204I). Their studies suggest a significant correlation between the fold resistance and the binding affinity of five anti-HBV agents: lamivudine, adefovir, entecavir, telbivudine and clevudine. Also, they analyzed different binding modes for synthetic nucleoside analogue drugs as well as natural nucleosides. Although their studies may not fully explain the difference of quantitative binding affinity, they showed detailed resistance mechanisms for anti-HBV drugs against wild-type and mutant HBV. 

Adefovir is active against wild-type and lamivudine-resistant strains of HBV [73]. In contrast to lamivudine therapy, adefovir is associated with delayed and uncommon selection of drug-resistant viruses [74]. Long-term treatment with adefovir dipivoxil leads to the rtN236T mutation, which displays reduced susceptibility to adefovir but remains sensitive to lamivudine [75]. Yadav V *et al*. [76] presented the molecular basis of the mechanism of adefovir-diphosphate against lamivudine-resistant mutants and its decrease in susceptibility for rtN236T HBV polymerase mutants. These molecular dynamics studies demonstrated that the rtN236T HBV polymerase mutation does not affect the binding affinity of the natural substrate (dATP), but it decreases the binding affinity of adefovir-diphosphate toward the rtN236T HBV polymerase. The lamivudine-resistant mutations, rtM204V and rtM204I, result in increased van der Waals contacts between adefovir-diphosphate and the mutated residues, which accounts for the better binding affinity of adefovir-diphosphate toward these mutants. The second lamivudine-related mutation, rtL180M, also results in increased van der Waals interactions between adefovir-diphosphate and the final residue of the primer strand, which accounts for the better binding affinity of adefovir-diphosphate in these mutants.

Warner *et al*. [48] determined the prevalence of rtL80V/I mutation in lamivudine-resistant HBV isolates and characterized the *in vitro* phenotype of the mutants. Although L80I increases sensitivity to lamivudine and imparts a replication defect, it enhances the *in vitro* replication of lamivudine-resistant (rtM204I) HBV. Molecular modeling revealed that Leu 80 does not interact directly with the enzyme’s substrates. Molecular models of HBV reverse transcriptase showed that, although Leu 80 is located distal to the enzyme’s dNTP binding pocket, substitution of isoleucine for leucine at this site partially restores replication efficiency by sufficiently changing the overall spatial alignment of other residues that are important for catalysis. These results imply that the presence of rtL80I decreases the enzyme’s affinity for both dNTPs and lamivudine triphosphate and that the decrease in affinity for lamivudine triphosphate is greater than the decrease in affinity for the natural substrate, dCTP.

As mentioned above, using the homology model structure of HBV polymerase, the amino acid changes resulting from mutations that give antiviral resistance can be mapped to functional regions to provide a better understanding of the molecular mechanism of resistance [51,71]. The HBV polymerase consists of four different domains: terminal protein, a space region, a catalytic RT domain and RNase H domain. We constructed and refined the model structure of HBV RT based on the homology to HIV-1 RT according to the reported method [72].

**Figure 1:**
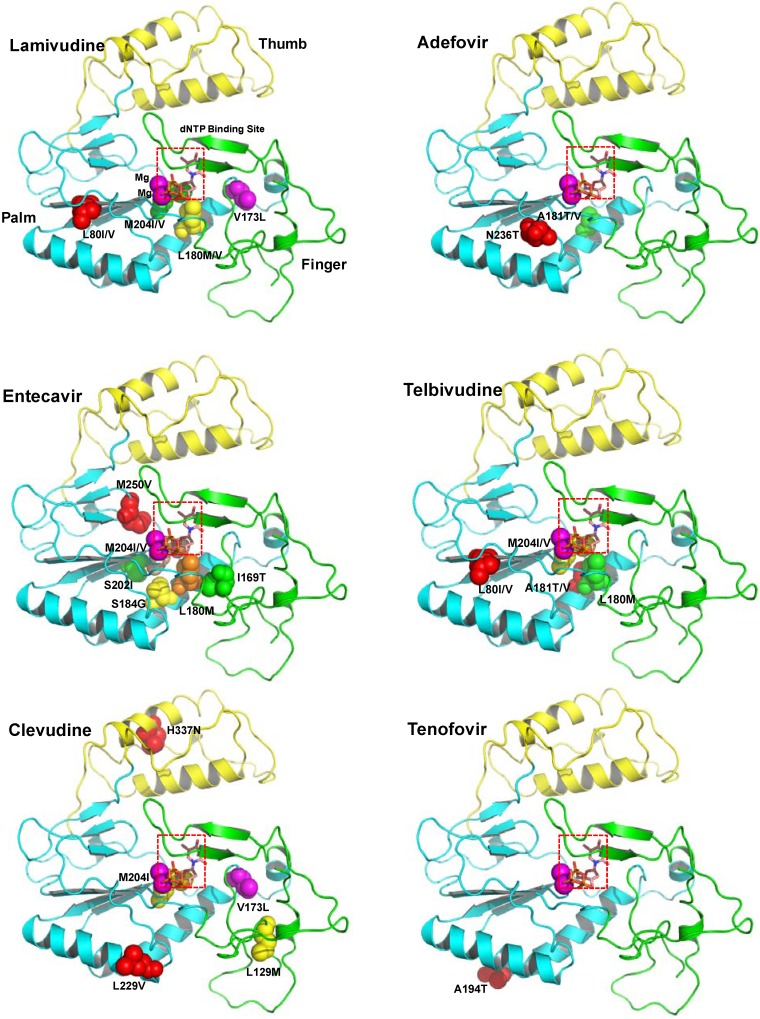
The ribbon diagram of homology model structure of HBV RT shows the location of the major mutations that confer resistance to clinically available six drugs. The HBV RT model structure was constructed and refined as previously reported [72]. HBV RT consists of three sub-domains: fingers (amino acid 1 to 49 and 90 to 172, in green), palm (amino acid 50 to 89 and 173 to 267, in cyan), and thumb (amino acid 268 to 351, in yellow). The locations of the mutations are indicated with the sphere model.

Figure 1 shows the location of the major mutations in RT that confer resistance to clinically available drugs. Detailed analysis of the model structure reveals that most of the drug-resistant mutations alter the conformation of at least one of the following sites: dNTP binding site, primer binding site, template binding site, dNTP incoming region, dNTP binding pocket, and magnesium ions binding site (highly conserved YMDD). 

## 4. Development of Anti-HBV Agents

The development of antiviral agents is compelled by the need to overcome life-threatening viruses such as HIV. Recently, greater understanding of viral life cycles and more advanced development tools have enabled us to discover and develop novel antiviral agents faster and more easily. The strategies and rationale in the design of antiviral drugs have been comprehensively reviewed [10]. The anti-HBV drugs in clinical trials and HBV inhibitors of antigens or replication are summarized in Table 2 and discussed in the following.

### 4.1. Drugs in clinical trials for HBV

There are several new drug candidates that are in clinical trials for the treatment of chronic hepatitis B. Lagociclovir valactate (known as MIV-210), a prodrug of 3′-fluoro-2′,3′-dideoxyguanosine with high oral bioavailability in humans and potent activity against hepatitis B virus, is currently in phase 1 and 2 clinical studies in Europe and Asia. MIV-210 is known to have activity against lamivudine-, adefovir-, and entecavir-resistant HBV variants. In the woodchuck model of HBV, treatment with MIV-210 at a dose of 20 mg/kg/day or 60 mg/kg/day was well tolerated and caused rapid and pronounced virological woodchuck hepatitis virus (WHV) DNA reduction as well as appreciable decreases in hepatic WHV cccDNA levels [77]. 

**Table 2:** Anti-HBV drugs in clinical trials.

Molecule	Structure	Brand name	Mechanism	Company	Year
Lagociclovir valactate	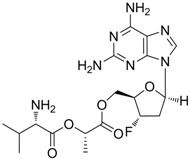	-	Nucleoside analogue/RT inhibitor/Prodrug	Medvir AB	Phase II 2005 (Sweden)
Elvucitabine	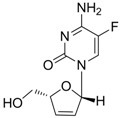	-	Nucleoside analogue/RT Inhibitor	Achillion Pharmaceuticals	2001 (US)
B-80380	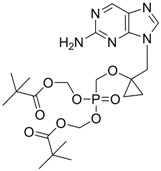	-	Nucleotide analogue/RT Inhibitor	LG Chem Ltd	Phase II 2003 (KOREA)
radefovir	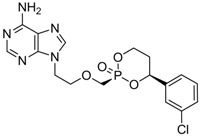	-	Nucleotide analogue/RT inhibitor/Prodrug	Metabasis Therapeutics	Phase II 2007 (US)
altorcitabine	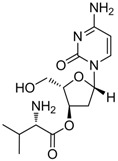		Nucleoside analogue/RT inhibitor/Prodrug	Idenix Pharmaceuticals/Novartis	Phase II 2003 (US)

Elvucitabine [2',3'-dideoxy-2',3'-didehydro-β-L(-)-5-fluorocytidine] is a nucleoside analogue RT inhibitor developed by Achillion Pharmaceuticals. *In vitro* studies and several phase I/phase II clinical trials have demonstrated that elvucitabine has significant potency against HIV. It also showed strong anti-HBV activity for the treatment of chronic HBV [78]. In the initial Phase 2 clinical trial for HIV, doses of 50 mg or greater per day were associated with an unacceptable reduction in patients’ white and red blood cells. Recently, the clinical trial was discontinued and the program was placed on hold while determination of the appropriate dosing regimen for elvucitabine was made for hepatitis B in the USA. 

LB-80380 is a nucleotide analogue RT inhibitor developed by LG Life Sciences, Ltd., in Korea for the treatment of HBV infection [79]. *In vitro* antiviral activity against wild-type and lamivudine-resistant mutants (rtM204I/V, rtL180M + rtM204I/V) showed similar EC_50_ values to adefovir [80]. LB80380 is currently in phase 2 clinical studies for chronic HBV patients in South Korea. The results of the clinical study suggest that LB80380 at doses of up to 240 mg for a period of 12 weeks is safe, well tolerated, and effective at reducing viral load in chronic hepatitis B patients with lamivudine-resistant virus [81,82].

Pradefovir, a selectively liver-activated prodrug of adefovir dipivoxil was developed by Metabasis Therapeutics. Pradefovir was developed to overcome the renal toxicity of adefovir dipivoxil and showed the delivery of adefovir and its metabolites to the liver with a 12-fold improvement in the liver/kidney ratio over adefovir dipivoxil [83].

Valtorcitabine, a well-absorbed prodrug of L-deoxycytidine, has also demonstrated potent suppression of serum HBV DNA in HBeAg-positive patients in a phase I/II study. Valtorcitabine was well-tolerated in all patient cohorts, with a safety profile comparable to that of placebo and synergistic with telbivudine for inhibiting HBV replication *in vitro* and in the woodchuck hepadnavirus model [84].

### 4.2. Novel non-nucleoside HBV inhibitors

As described above, all currently available anti-HBV drugs are nucleoside or nucleotide analogues and inevitably associated with the development of HBV resistance to drugs because they inhibit the RT by competing with the natural dNTPs. Therefore, agents that can interfere with other targets in the viral life cycle are strongly needed for combination therapies, such as those used for the treatment of HIV. In this regard, many research groups are dedicated to developing non-nucleoside anti-HBV agents that exert different modes of action than nucleoside or nucleotide inhibitors. In this section, we divided the HBV inhibitors into two subgroups according to the mode of action: HBV inhibitors targeting viral antigens or replication. Table 3 summarizes the characteristics of non-nucleoside HBV inhibitors that target viral antigens. 

Zhang *et al*. [85,86,87] synthesized a series of alisol A derivatives and evaluated their anti-HBV activities and cytotoxicities *in vitro* using lamivudine as a positive control. Their results demonstrate that simple modifications to the parent structure of alisol A resulted in inhibitory potency against secretion of hepatitis B surface antigen (HBsAg), such as compound **1** (Table 1). Ellagic acid isolated from *Phyllanthus urinaria* (compound **2**) showed unique anti-HBV activities. Ellagic acid did not inhibit HBV replication or HBsAg secretion; rather, it blocked HBeAg secretion very effectively in HepG2 2.2.15 cells [88]. Compound **3** is a selective, potent inhibitor of secretion of both subviral and DNA-containing viral particles [89]. It did not affect the viral replication and HBeAg secretion. Further analysis showed that compound **3** is a specific inhibitor of HBsAg secretion. Although the exact mechanism of action of compound **3** remains to be elucidated, these observations suggest that its effects occur through direct action on the HBV viral and subviral particles themselves, the antigens that they contain, or the cellular processes or factors that are uniquely required for the secretion of these particles. Su *et al*. [90] found that the natural pyranocoumarin derivatives (compound **4**) suppressed the secretion of HBsAg in HepA2 cells and also showed cytotoxic activity against four human cancer cell lines (A549, MCF7, KB, and KB-VIN). The most interesting result in the cytotoxicity assay was that compound **4** showed significant activity only against KB-VIN cells, the multi-drug resistant cell line; on the other hand, they showed marginal or no activity against the other three cell lines. 

**Table 3:** Summary of non-nucleoside HBV inhibitors that target viral antigens.

Compound (Target)	Structure	Activity	Mechanism of Action	Reference
**1** (HBsAg, HBeAg)	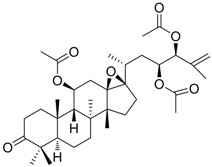	CC_50 _: >2.6mM HBsAg(IC_50_): 0.024 mM HBeAg(IC_50_): 0.028 mM	Inhibition of HBsAg and HBeAg secretion	[85]
**2** (HBeAg)	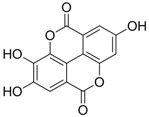	IC_50_: 0.07 μg/mL	Inhibition of HBeAg secretion in HepG2 2.2.15 cell line	[88]
**3** (HBsAg)	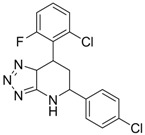	EC_50_: 1.5 μM	Inhibition of HBsAg secretion	[89]
**4** (HBsAg)	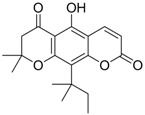	EC_50_: 1.14 μM	Inhibition of HBsAg secretion	[90]
**5** (Virion secretion)	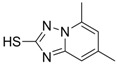	IC_50_: 0.12 μM	Inhibition of the interaction between core and surface protein	[91]
**6** (Virion secretion)	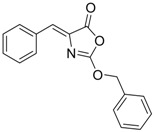	IC_50_: 5.4 μM	Inhibition of the interaction between core and surface protein	[91]
**7** (Capsid formation)	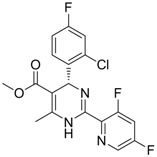	IC_50_: 0.05 μM	Inhibition of replication by nucleocapsid depletion	[92]

Using chemical library screening, Asif-Ullah *et al*. [91] identified compounds **5** and **6**, which inhibit the interaction between the core protein and PreS region of the surface protein. These compounds prevented the production of HBV particles from transiently HBV-producing HuH7 cells. The IC_50_ values of these compounds for inhibition of HBV production in HuH7 cells were 0.12 and 5.4 μM for compound **5** and compound **6**, respectively. Although these compounds showed promising anti-HBV activity, the detailed mechanism of how the compounds interfere with the surface and core protein interactions requires further investigation.

Compound **7** inhibits HBV nucleocapsid maturation leading to potent *in vitro* and *in vivo* antiviral activity [92]. It displayed a highly specific antiviral principle, namely, inhibition of capsid formation, concomitant with a reduced half-life of the core protein. This compound may become a valuable addition to future therapy in light of its specific mechanism of action. *In vitro* cell studies as well as HBV transgenic mice models showed a suitable preclinical pharmacokinetic and toxicology profile with high efficacy.

The characteristics of non-nucleoside HBV inhibitors targeting viral replication are summarized in Table 4. The phenylpropenamide derivatives are potent inhibitors of *in vitro* HBV replication in stably or transiently transfected HepG2 cells [93,94]. These compounds showed no significant differences in sensitivity between wild-type and nucleoside analogue-resistant (rtL180M, rtM204I, and rtL180M + rtM204V) HBV. Although the mechanism of action of compound **8** is not clearly understood, it appears to be independent of interference with the RNA- or DNA-dependent activities of the HBV polymerase. Recently, Feld and colleagues found that compound **8** decreased the amount of RNA-containing cytoplasmic HBV core particles, suggesting that this compound blocks HBV replication at the level of viral RNA packaging [95]. 

A series of non-nucleoside ethyl 6-hydroxyquinoline-3-carboxylate derivatives (compound **9** in Table 4) were synthesized and evaluated for anti-HBV activity in HepG2.2.15 cells [96]. Compound **9** derivatives inhibited the expression of viral antigens HBsAg or HBeAg at low concentration and displayed excellent intracellular inhibitory activity and selectivity towards the replication of HBV DNA. Of these ethyl 6-hydroxyquinoline-3-carboxylate derivatives, compound **9** was the most potent and highly specific inhibitor of HBV DNA replication in cell culture. 

**Table 4:** Summary of non-nucleoside HBV inhibitors that target viral replication.

Compound	Structure	Activity	Mechanism of Action	Reference
**8**	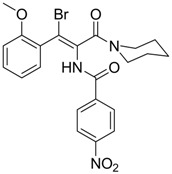	IC_50_: 2.4 μM in HepG2 cells	Inhibition of replication by blocking RNA packaging	[93,94,95]
**9**	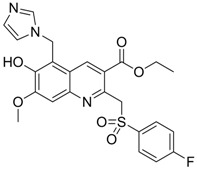	IC_50_: 4.7 μM (Replication) IC_50_: 26.2 μM (HBsAg) IC_50_: 98.1 μM (HBeAg) in HepG2 2.2.15 cells	Inhibition of replication, HBsAg and HBeAg secretion	[96]
**10**	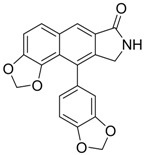	IC_50_: 0.08 μM in HepG2 2.2.15 cells	Inhibition of replication by down-regulation of HNF-3 and 4	[97,98]
**11**	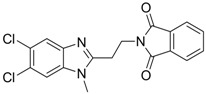	IC_50_: 0.9 μM in HepG2 cells CC_50_: >1000 μM	Inhibition of replication	[99]
**12**	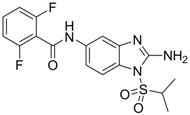	IC_50_: 0.7 μM CC_50_: 192 μM in HepG2 2.2.15 cells	Inhibition of replication	[100]
**13**	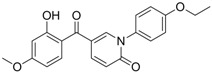	IC_50_: 0.206 μM CC_50_: >109.6 μM in HepG2 cells	Inhibition of replication	[101]
**14**	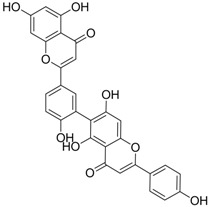	IC_50_: 0.25 μM in HepG2 2.2.15 cells	Inhibition of replication	[102]
**15**	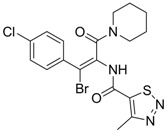	IC_50_: 3.59 μg/mL in HepG2 2.2.15 cells	Inhibition of replication	[103]
**16**	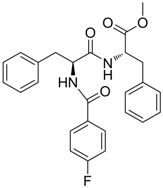	IC_50_: 1.40 μM in HepG2 2.2.15 cells	Inhibition of replication, HBsAg and HBeAg secretion	[104]
**17**	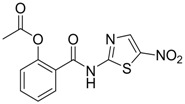	EC_50_: 0.12 μM in HepG2 2.2.15 cells	Inhibition of replication	[105]
**18**	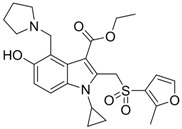	IC_50_: 1.52 μg/mL in HepG2 2.2.15	Inhibition of replication	[106]
**19**	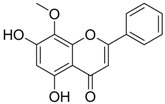	IC_50_: 0.57 μg/mL in DHBV replication. IC_50_: 4.0 μg/mL (HBsAg, HBeAg)	Inhibition of replication, HBsAg and HBeAg secretion	[107]
**20**	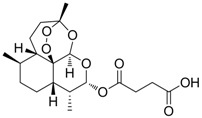	IC_50_: 2.3 μM (HBsAg) IC_50_: 0.5 μM (replication)	Inhibition of replication and HBsAg secretion	[108]
**21**	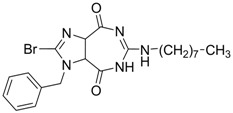	EC_50_: 1.7 μM, CC_50_: 286 μM in HepG2 2.2.15 cells	Inhibition of virion secretion and replication	[109]
**22**	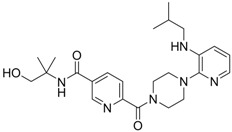	IC_50_: ~0.01 μg/mL	Inhibition of RT	[110]
**23**	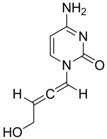	IC_50_:0.08 μM in HepG2 2.2.15	Inhibition of replication	[111]

Helioxanthin and its derivative 5-4-2 (compound **10**), a novel class of compounds, were reported to have potent anti-HBV activities in HepG2.2.15 cells, with EC_50_s of 1 and 0.08 μM, respectively [97]. The lamivudine-resistant rtL180M/M204V double mutant HBV strain was also sensitive to helioxanthin and compound **10**. This class of compounds not only inhibited HBV DNA, but also decreased HBV mRNA and core protein expression. A recent study has demonstrated that compound **10** suppresses HBV replication by post-transcriptional down-regulation of critical transcription factors, which are essential for HBV gene expression, hepatocyte nuclear factor (HNF) 4 and 3 [98]. Compound **10** was a potential novel anti-HBV agent that possesses a different mechanism of action from existing therapeutic drugs. 

Compounds **11** ~ **23** in Table 4 and their derivatives were synthesized and evaluated for their anti-HBV activity and cytotoxicity *in vitro* (see references in Table 4). All compounds exhibited strong activity against HBV replication with low cytotoxicity *in vitro.* However, the stages of the viral life cycle or the targets involved in their mechanisms of action have not been determined. Further studies on the mechanisms of action and preclinical evaluations using animal models are therefore needed to describe this novel class of anti-HBV drug candidates.

### 4.3. Virtual screening, modeling and rational drug design

Virtual screening is an efficient method for identifying drug candidates *in silico* from large chemical compound databases. Virtual screening, particularly receptor-based or ligand-based virtual screening, has emerged as a reliable, inexpensive method for identifying lead compounds. Advances in computational techniques have enabled virtual screening to be a powerful tool in the drug discovery process. Its usefulness has been verified by current applications in which hit and lead identifications against various disease targets were successfully retrieved (reviewed in reference [112]). There have been great advances in molecular modeling techniques that have resulted in the excavation of a large number of potential therapeutic drugs that are relevant to biological functions. The growth in the number of potential target molecules has increased the demand for reliable target validation as well as for technologies that can rapidly identify optimal lead candidates. 

Antiviral compounds have also recently been successfully identified by virtual screening. Many laboratories have investigated inhibitors of HIV-1 and Hepatitis C virus (HCV) using various targets and virtual screening methods [113,114,115,116,117,118]. Applications and examples of pharmacophore-based antiviral drug design are comprehensively reviewed recently [112]. 

However, unfortunately, there has been no report of an HBV inhibitor study by virtual screening methods. This may partly be due to the lack of an X-ray crystal structure of HBV polymerase, which is needed to obtain reliable virtual screening results. Recently, a pharmacophore model of the synthesized compounds was proposed by the genetic algorithm similarity program (GASP) program [119] to give useful information for further structural modification [101]. Lv and colleagues synthesized the 2-pyridone derivatives and tested for their *in vitro* anti-HBV activity [101]. Because the target of 2-pyridone derivatives is unknown, the construction a pharmacophore model would be useful for further structural optimization and could also be used in virtual screening and for identifying new lead structures. 

The refined homology model structure of HBV based on the crystal structure of HIV-1 RT has successfully demonstrated the molecular mechanism of drug resistance to corresponding drugs [120]. The homology models provide mechanistic insight into HBV resistance that is consistent with the biological studies, although it still lacks the validation of direct structural data. Until the 3D structure of HBV polymerase is defined, the homology model structure will serve as a useful tool for better understanding the molecular mechanism of replication and resistance and for the investigation of anti-HBV lead compounds. 

### 4.4. Targets for future drugs

As described earlier, all current anti-HBV drugs are RT inhibitors of nucleoside or nucleotide analogues, leading to a high possibility of developing drug-resistant HBV. Treatment with multiple drugs having different mechanisms of action is therefore needed to improve viral clearance and prevent resistance. Thus, small molecule inhibitors that target the viral life cycle proteins other than polymerase are strongly needed for combination therapy. 

RT inhibitors suppress viral replication very efficiently but do not eliminate the existing virus and cccDNA. If the episomal cccDNA in hepatocytes is not eliminated, HBV can rebound if the conditions are suitable for virion production. Recently, a fundamental understanding of the regulation of cccDNA has been elucidated by some groups [121,122,123,124]. The main features of their findings on cccDNA regulation are the involvement of HBx and the epigenetic control of cccDNA-bound histones and cccDNA itself. These efforts will allow development of future drugs that can control the biogenesis and regulation of cccDNA, the ultimate goal of HBV eradication. 

The inhibitors that have been reported to exert specific and potent anti-HBV activity with non-polymerase viral targets *in vivo* and *in vitro* warrant further evaluation in clinical trials. Small molecule inhibitors such as compound **7** (in Table 3) and compound **8** (in Table 4) efficiently block the nucleocapsid maturation [92] and pregenomicRNA encapsidation [93,94,95], respectively. HBV entry inhibitors that utilized the pre-S1 peptides, which are essential for virus entry into the hepatocytes, successfully prevent viral infection [125]. Introduction of intact or modified small-interfering RNA produces strong and persistent anti-HBV activity *in vivo* and *in vitro* [126,127,128]. The chemical inhibitors (compounds **5** and **6** in Table 3), which block the interaction between core and surface protein, exert strong anti-HBV activity [91]. The inhibition of this interaction will eventually block the maturation of virion assembly and virion secretion. Along with these inhibitors, materials that can block viral and host targets that are essential for HBV life cycle, such as HBx [129], HNF [98] and unidentified HBV receptor(s) [130], are candidates for future drugs against HBV. 

## 5. Conclusions

Treatment for chronic hepatitis B patients depends on anti-HBV drugs. Even though the currently approved anti-HBV drugs, nucleos(t)ide analogues, show potent and fast antiviral response, several problems remain to be solved. These include the development of resistance and the side effects such as myopathy, which is induced by mitochondrial damage. Based on advances in the development of antiviral agents along with newly discovered drug candidates and combination therapy, resistance should not be a great concern in the near future. However, combination therapy for effective control of HBV requires the development of novel drugs that have different mechanisms of action. 

The mitochondrial damage is mainly due to the high affinity of nucleos(t)ide RT inhibitors for mitochondrial DNA polymerase gamma. Therefore, alleviation of side effects should be considered in the development of future nucleos(t)ide drugs. In this regard, the fine crystal structure of polymerase gamma was reported recently [131,132]. The elucidation of the polymerase gamma structure establishes a foundation for understanding the molecular basis of the toxicity of anti-retroviral drugs targeting HBV and HIV and the cause of cellular toxicity induced by some antiviral nucleoside analogs. Eventually, these fundamental studies in conjunction with advanced drug development tools will provide valuable information for the development of novel drugs without side effects.
